# Structure, Conductivity, and Sensor Properties of Nanosized ZnO-In_2_O_3_ Composites: Influence of Synthesis Method

**DOI:** 10.3390/mi14091685

**Published:** 2023-08-29

**Authors:** Mariya I. Ikim, Vladimir F. Gromov, Genrikh N. Gerasimov, Elena Y. Spiridonova, Anastasiya R. Erofeeva, Kairat S. Kurmangaleev, Kirill S. Polunin, Olusegun J. Ilegbusi, Leonid I. Trakhtenberg

**Affiliations:** 1 N.N. Semenov Federal Research Center for Chemical Physics of RAS, Moscow 119991, Russiaf7033@mail.ru (K.S.K.); kpolunin62@gmail.com (K.S.P.); 2 Department of Mechanical and Aerospace Engineering, University of Central Florida, Orlando, FL 32816, USA; 3 Chemical Faculty, Lomonosov Moscow State University, Moscow 119991, Russia

**Keywords:** zinc oxide, indium oxide, nanostructured composite, synthesis method, impregnation, hydrothermal method, particle size, conductivity, sensor response, hydrogen

## Abstract

The influence of the method used for synthesizing ZnO-In_2_O_3_ composites (nanopowder mixing, impregnation, and hydrothermal method) on the structure, conductivity, and sensor properties is investigated. With the nanopowder mixing, the size of the parent nanoparticles in the composite remains practically unchanged in the range of 50–100 nm. The impregnation composites consist of 70 nm In_2_O_3_ nanoparticles with ZnO nanoclusters < 30 nm in size located on its surface. The nanoparticles in the hydrothermal composites have a narrow size distribution in the range of 10–20 nm. The specific surface of hydrothermal samples is five times higher than that of impregnated samples. The sensor response of the impregnated composite to 1100 ppm H_2_ is 1.3–1.5 times higher than the response of the mixed composite. Additives of 15–20 and 85 wt.% ZnO to mixed and impregnated composites lead to an increase in the response compared with pure In_2_O_3_. In the case of hydrothermal composite, up to 20 wt.% ZnO addition leads to a decrease in response, but 65 wt.% ZnO addition increases response by almost two times compared with pure In_2_O_3_. The sensor activity of a hydrothermal composite depends on the phase composition of In_2_O_3_. The maximum efficiency is reached for the composite containing cubic In_2_O_3_ and the minimum for rhombohedral In_2_O_3_. An explanation is provided for the observed effects.

## 1. Introduction

The electrical resistance of nanostructured semiconductor metal oxides such as SnO_2_, In_2_O_3_, CeO_2_, and Fe_2_O_3_ changes in the presence of various gases, including H_2_, CO, CH_4_, NH_3_, O_3_, ethanol, acetone and aromatic compounds. This makes it possible to use such oxides as sensors for monitoring environmental composition. It has been shown that sensors based on nanoparticles of semiconductor metal oxides with particle sizes of about 100 nm have the greatest efficiency [1].

Particles of such a size generally have a large specific surface area and high energy. The presence of such surface energy in the system contributes to the sensor phenomenon, allowing it to overcome the activation energy of reactions [2]. It is also important that materials consisting of nanoscale particles acquire new physicochemical and operational characteristics. It should also be noted that the specific surface area and the surface-to-volume ratio of the nanostructured material increase with the transition to smaller particles. This is one major factor in favor of using nanoscale materials to create conductometric sensors.

Sensors based only on one metal oxide have insufficiently high efficiency and selectivity in the detection of multicomponent gas mixtures. It has been established that in order to obtain effective sensors, the systems must comprise a mixture of oxides, one of which has a high concentration of conduction electrons and the other a high catalytic activity in oxidation and dissociation reactions [3,4,5,6].

The conductive and sensor properties of binary metal oxide composites largely depend on the synthesis method, which can affect the type of interaction between the nanoparticles of the system (see, for example, Refs. [7,8,9,10,11,12]). The most important types of interaction affecting the sensor response in binary nanosystems include the transfer of atomic oxygen between catalytically active and electron-rich nanoparticles, as well as the introduction of ions of particles of one oxide into the other component [13,14,15,16].

One of the metal oxides widely used to produce sensors is In_2_O_3_, and *n*-type semiconductor with a high concentration of conduction electrons and a band gap of 2.8 eV [17,18]. Another oxide is the well-known photocatalyst ZnO, which has a wide band gap of 3.2 eV and excellent chemical and thermal stability [17]. In addition, it is an inexpensive and non-toxic material. The solubility limit of Zn in In_2_O_3_ is close to 1 at.% [18]. As a result of heat treatment of a mixture of zinc and indium oxides, a solid substitution solution with a composition of up to 0.5 at.% Zn is formed, which, upon increase in zinc oxide concentration, transitions into a solid solution with an admixture of zinc exceeding 0.5 at.%. The introduction of 0–5% Zn into indium oxide leads to a slight increase in both the parameter “a” of the crystal lattice and the size of the crystallites, as well as a decrease in the band gap [19].

The efficiency of an In_2_O_3_-based sensor can be significantly increased by the addition of zinc oxide to the sensitive layer to improve the gas-sensitive properties in response to various compounds [20,21,22]. When exposed to radiation in the visible or ultraviolet ranges, sensors based on ZnO-In_2_O_3_ heterostructures are capable of detecting various gases even at room temperature [23,24]. In the vast majority of studies on ZnO-In_2_O_3_ heterostructures, various additives of zinc oxide are usually considered, while there is no justification for choosing the composition of such composites. Such systems are mainly used for the detection of ethanol [21,25,26,27,28] or triethylamine [29,30,31].

However, data on the optimal concentration of zinc oxide in ZnO-In_2_O_3_ composites used in gas sensors are contradictory and depend on many factors. In this regard, the ZnO-In_2_O_3_ composites were synthesized in the present study in the entire concentration range from 0 to 100% zinc oxide, and the influence of the method of synthesizing ZnO-In_2_O_3_ composites on their phase composition, structure, conductivity, and sensor properties during hydrogen detection was studied. Three synthesis methods were investigated: mixing of commercial ZnO and In_2_O_3_ nanopowders, impregnation of indium oxide nanopowder with zinc salt and subsequent formation of ZnO nanoclusters during heat treatment, and the hydrothermal method using aqueous solutions of zinc and indium nitrates.

## 2. Experiments Performed

Systems of ZnO-In_2_O_3_ composites were synthesized using three methods. In the first method, the composites were obtained using commercial nanopowders of the corresponding metal oxides In_2_O_3_ (AnalaR grade, 99.5%, BDH/Merck Ltd., Lutterworth, Leicestershire, UK) and ZnO (99.9%, Sigma–Aldrich Chemical Co., Gillingham, UK), the particle sizes of which ranged from 50 to 100 nm. The necessary amounts of metal oxide nanopowders were thoroughly ground with water in a ball mill for 1–2 h. This synthesis method produced binary metal oxide nanocomposites containing separate crystals of each of the constituent metal oxides [14].

To obtain composites by the impregnation method, a commercial indium oxide nanopowder was placed in an aqueous solution of zinc nitrate Zn(NO_3_)_2_·6H_2_O (purity ≥ 99%) and kept at room temperature for 24–48 h. As a result of such prolonged treatment, the salt solution completely and evenly wetted the surface of indium oxide. Concurrently, the zinc nitrate molecules were adsorbed not only on the surface of the powder but also diffused into the near-surface layer. Subsequent removal of water at temperatures of about 70–80 °C and heating the sample for several hours to 300 °C resulted in an impregnated composite consisting of indium oxide particles with small particles of zinc oxide embedded on their surfaces [8].

In the preparation of ZnO-In_2_O_3_ composites by the hydrothermal method, indium nitrate In(NO_3_)_3_·4H_2_O (≥99.5%) and zinc nitrate Zn(NO_3_)_2_·6H_2_O (≥99%) were used as precursors. For hydrolysis, aqueous solutions of these salts were kept in a medium with a large excess NaOH, which allowed the pH value of the medium to be maintained constant during the entire process. The zinc and indium hydroxides formed were separated in a centrifuge at a speed of 4000 rpm for 15 min and then thoroughly washed with distilled water. For hydrothermal treatment, the resulting product was placed in a 25 mL Teflon-lined stainless-steel autoclave and kept at a temperature of 180 °C for 1 h. To convert the hydroxides processed in the autoclave into the corresponding individual oxides or the ZnO-In_2_O_3_ composite, they were heated in air at 500 °C until a constant resistance was achieved. The three methods described above were used to prepare samples of ZnO-In_2_O_3_ composites containing from 5 to 85 wt.% zinc oxide. The samples were subsequently used for microstructural and compositional characterization and the measurement of conductivity and sensor properties.

The phase composition, structure, and morphology of the composites were determined by X-ray diffraction (XRD), transmission and scanning electron microscopy (TEM and SEM), energy dispersive X-ray spectroscopy (EDX) and low-temperature gas adsorption. XRD spectra were recorded on a Rigaku Smartlab SE X-ray diffractometer using Cu Kα radiation with a wavelength of 1.5406 Å. The average size of nanoparticles was determined from XRD spectroscopy data according to the Debye-Scherrer formula: D = 0.9λ/(βcosθ), where λ is the wavelength of X-ray radiation, β is the half-width of the peak, and θ is the diffraction angle corresponding to this reflex.

The morphology of particles in the composites and the distribution of metal ions between the components of the composite were determined by SEM on the Prisma E device and TEM and EDX, on the Tecnai Osiris FEI equipped with energy-dispersion analysis The dot is system. The specific surface area and average pore size were determined by low-temperature nitrogen adsorption on the NOVA Series 1200e Quantachrome (USA) device.

To determine the conductivity and sensor properties, the synthesized composites were mixed with distilled water. The resulting paste was applied to a special chip equipped with a platinum heater and contacts for recording the corresponding electrophysical characteristics, gradually raising the temperature to 550 °C until a constant resistance of the resulting film was achieved.

The sensor resistance change was recorded using the Digital Multimeter Keysight, the signal from which was transmitted to the computer. The installed computer program was used to obtain the kinetic curve of the change in resistance. The sensor responses S = R_o_/R_g_ were determined from these data, where R_o_ is the initial resistance of the sensor (before the introduction of the analyzed mixture) and R_g_ is the minimum value of the sensor resistance achieved after the introduction of the analyzed gas.

The sensor response of the prepared films to H_2_ was studied using the device developed in the temperature range of 280 to 520 °C. The accuracy of maintaining the temperature was within 1 °C. The chip with the applied sensitive layer was located in a special chamber with a volume of about 1 cm^3^ into which purified air or a calibration gas mixture containing 0.1% H_2_ was supplied. The gases were pumped through the chamber at a rate of 200 mL/min.

## 3. Results and Discussions

The structure, morphology, conductivity, and sensor response to hydrogen of ZnO-In_2_O_3_ composites obtained by the three approaches described in the previous section were determined by modern physicochemical methods. The analysis of these data enables the determination of the effect of the synthesis method on the phase composition, structural characteristics, as well as the conductive and sensor properties of the composites.

### 3.1. Structural Characteristics of ZnO-In_2_O_3_ Composites

**Figure 1:**
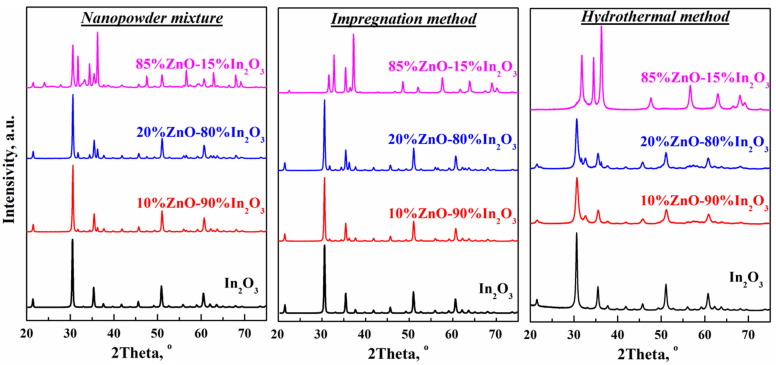
XRD spectra of ZnO-In_2_O_3_ composites obtained by mixing powders, impregnation methods, and hydrothermal methods.

The XRD spectra of ZnO-In_2_O_3_ composites obtained by the three synthesis methods are presented in Figure 1. Analysis of the XRD spectra of composites obtained by mixing powders and impregnation methods shows that the two synthesis methods lead to the formation of two-phase composites—ZnO-In_2_O_3_. Diffraction peaks at 21.5, 30.6, 35.5, 37.7, 41.8, 45.7, 51.0, 55.9, and 60.7° correspond to planes (211), (222), (400), (411), (332), (431), (440), (611), and (622) of indium oxide, respectively. Peaks at 31.7, 34.1, 36.1, 47.3, 56.5, 62.7, and 67.7° can be attributed to the (100), (002), (101), (102), (110), (103), and (112) planes of the oxide zinc, respectively. For composite ZnO-In_2_O_3_ obtained by mixing powders and impregnation methods, all diffraction peaks correspond to the cubic structure of bixbyite In_2_O_3_ (JCPDS no. 06-0416) and hexagonal ZnO with a wurtzite structure (JCPDS no. 36-1451).

On the XRD spectra of ZnO-In_2_O_3_ composites synthesized by the hydrothermal method, peaks were recorded that relate to both the cubic and rhombohedral phases of In_2_O_3_ (Figure 1). The data were compliant with JCPDS #22-0336. A similar pattern was observed in the ZnO-In_2_O_3_ and In_2_O_3_-ZnO systems synthesized by the two-step chemical precipitation methods [27]. There are no diffraction peaks of ZnO in the XRD spectra of hydrothermal composites containing up to 20% ZnO. This may be due either to the introduction of Zn ions into the crystal lattice of indium oxide or the formation of an X-ray amorphous oxide phase. Our previous studies have shown that SnO_2_-In_2_O_3_ composites also exhibit this behavior [10].

The rhombohedral phase of In_2_O_3_ is indexed on XRD spectra only at concentrations of 10, 20, and 40% ZnO in the composites. The formation of the rhombohedral phase of In_2_O_3_ occurs due to the partial dissolution of Zn ions, which have a smaller size than In ions, in the lattice of basic cubic In_2_O_3_ crystals. In addition, this phase has concentrations of 15, 22, and 9 wt.% for 10, 20, and 40% ZnO, respectively. Interestingly, the concentration of the rhombohedral phase of indium oxide is maximum in hydrothermal composites containing 20% ZnO. Also, only the cubic phase of indium oxide is observed in the composites with a 65% ZnO concentration. It has been shown that the ratio between the rhombohedral and cubic phases of indium oxide can be controlled by the concentration of zinc in structures synthesized by a simple solvothermic method [32].

In the spectra of ZnO-In_2_O_3_ composites containing 20 to 85% ZnO, peaks appear corresponding to reflexes of ZnO with a wurtzite structure (JCPDS no. 36-1451). The compositions of zinc oxide in these composites, calculated by the Rietveld method, are 9.7, 29.5, 64.9, and 100%. Note that the concentration calculated from the XRD data corresponds to that used for synthesis only for the 65%ZnO-35%In_2_O_3_ composite. It can be assumed that the ZnO-In_2_O_3_ heterostructure is formed only at this concentration. At other concentrations of zinc, one component is introduced into the structure of another. The absence of any extraneous peaks indicates the high purity and crystallinity of the samples synthesized by the three methods used in this study.

In contrast to mixed and impregnated composites, the main peak of indium oxide characterizing the plane (222) in hydrothermal samples is located in the region of large angles compared with the situation in pure In_2_O_3_ (see Figure 1). The observed displacement of the indium oxide peak (222) to large angles indicates that zinc ions are embedded in the crystal lattice of indium oxide. In addition, the lattice parameter in hydrothermal composites with concentrations ranging from 0 to 65% ZnO decreases from 1.013 nm to 1.089 nm. This is because the size of the ionic radius of indium (0.81 Å) exceeds the radius of zinc (0.74 Å). It should be noted that in the 65%ZnO-35%In_2_O_3_ composite, the In_2_O_3_ lattice parameter reverts to the value of the initial indium oxide.

The particle size of zinc oxide calculated by the Debye-Scherrer formula is 45–50 nm in mixed composites and 10–30 nm in impregnated samples. The size of these nanoparticles in mixed composites does not depend on the zinc oxide concentration. On the contrary, the particle size of ZnO in impregnated composites decreases from 30 to 10 nm as its concentration in the mixture with indium oxide increases. It is probable that with an increase in the concentration of zinc salt, a large number of crystallization centers appear in the process of impregnation, which prevent the growth of embryos and, consequently, the formation of large crystals. In addition, the size of indium oxide nanoparticles in samples prepared by these two methods was calculated from the data from X-ray diffraction analysis. Calculations have shown that the size of indium oxide particles is approximately 70 nm and does not depend on the zinc oxide concentration in such samples.

According to XRD data, an increase in the concentration of zinc oxide in hydrothermal composites is accompanied by a decrease in the size of indium oxide nanoparticles from 25 to 9 nm. This result indicates that during synthesis, Zn atoms replace In atoms in the lattice, which creates deformations in the composites [33]. Thus, the introduction of Zn ions into the In_2_O_3_ structure hinders crystal growth. In composites containing from 20 to 85 wt.% zinc oxide, the particle size of ZnO is almost three times larger than that of In_2_O_3_ and decreases from 72 to 25 nm. The observed difference in the sizes of nanoparticles of zinc oxide and indium oxide may indicate that zinc oxide completely covers the surface of indium oxide during the formation of crystalline zinc oxide in hydrothermal composites, resulting in the formation of a core-shell structure.

**Figure 2:**
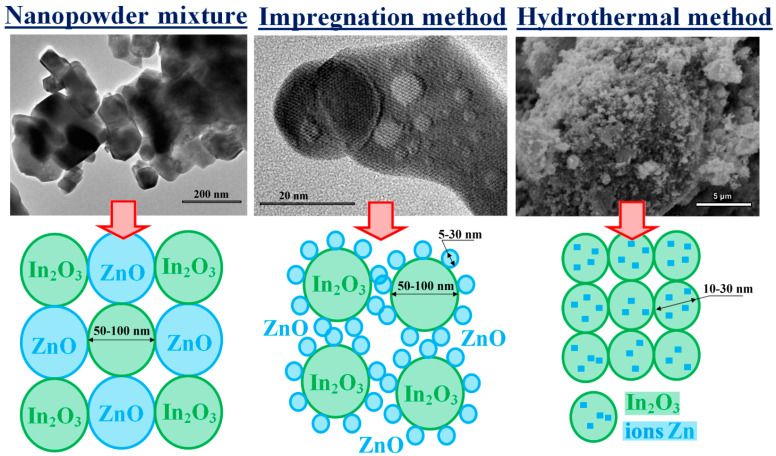
TEM images of mixed and impregnated composites containing 20% ZnO and SEM images of a hydrothermal sample of 5%ZnO-95%In_2_O_3_. Schematic representation of the structure of ZnO-In_2_O_3_ composites synthesized by different methods.

According to TEM data, the mixed ZnO-In_2_O_3_ nanocomposites are a mixture of nanocrystals of the corresponding oxides, have a diverse shape, and are characterized by a wide particle size distribution from 50 to 100 nm, which practically coincides with the particle sizes of the initial commercial nanopowders (Figure 2). Another picture is observed for impregnated composites. As a result of impregnation of indium oxide with zinc nitrate and subsequent heat treatment, small ZnO nanoparticles of almost spherical shape appear on the surface of relatively large indium oxide nanocrystals (up to 100 nm) (Figure 2).

The particle size varies from 5 to 30 nm, depending on the zinc oxide concentration in the composite. Thus, in impregnated samples of ZnO-In_2_O_3_, the particle size is approximately 20–30 nm for zinc oxide concentrations of 10–60%, but 5–10 nm for ZnO concentrations of more than 60%. According to XRD data, zinc ions are introduced into the indium oxide structure in hydrothermal composites. This result is confirmed by SEM and EDX data. The 5%ZnO-95%In_2_O_3_ hydrothermal composite consists of indium oxide particles with an inhomogeneous, loose structure in which zinc ions are fairly evenly distributed (Figure 2).

**Figure 3:**
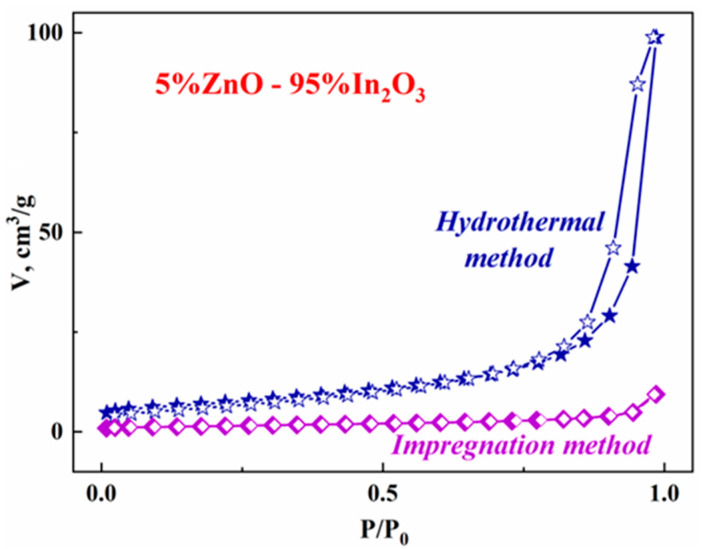
Adsorption (filled symbols) and desorption (empty symbols) isotherms of N_2_ at a temperature of 77 K for impregnated and hydrothermal composites containing 5% zinc oxide.

To analyze the specific surface area and porosity of the synthesized samples, nitrogen adsorption-desorption experiments were carried out at a temperature of 77 K (see Figure 3). According to the IUPAC classification, the adsorption-desorption isotherms of the hydrothermal composite have a type IV shape with a hysteresis loop H3. Such a shape is typical of the mesoporous nature of the samples. At the same time, the desorption and adsorption isotherms for samples obtained by mixing nanopowders and the impregnation method (Figure 3) have a form characteristic of macroporous samples.

The surface area calculated by the BET method was 25 m^2^/g for hydrothermal samples and five times less for mixed and impregnated samples (Figure 3). To study the mesoporous structure of hydrothermal samples, we used the BJH method for the desorption branch of the isotherm. The pore size of hydrothermal samples is 18–20 nm. It should be noted that the introduction of zinc oxide leads to an increase in the specific surface area of the sample, the volume of pores, and the pore size. The largest specific surface area (32 m^2^/g) is observed for the hydrothermal composite containing 65% ZnO. A higher specific surface area and a smaller average pore size of hydrothermal composites compared with mixed and impregnated composites facilitate the adsorption of O_2_ and the analyzed gas, which should lead to an acceleration of the response-recovery processes during gas detection.

### 3.2. Conductivity of ZnO-In_2_O_3_ Composites

The temperature dependence of the conductivity in air of composites obtained by the various methods investigated exhibits the same characteristics. In the range of 300 to 520 °C, the conductivity of all synthesized systems increases with rising temperature, which is typical for *n*-type semiconductors. In all the cases considered, the temperature dependence of the resistance of synthesized composites is not described by the Arrhenius law. The deviation from the Arrhenius law is obviously due to the fact that an increase in temperature leads to the desorption of chemisorbed active oxygen particles located on the surface of the sensitive layer.

**Figure 4:**
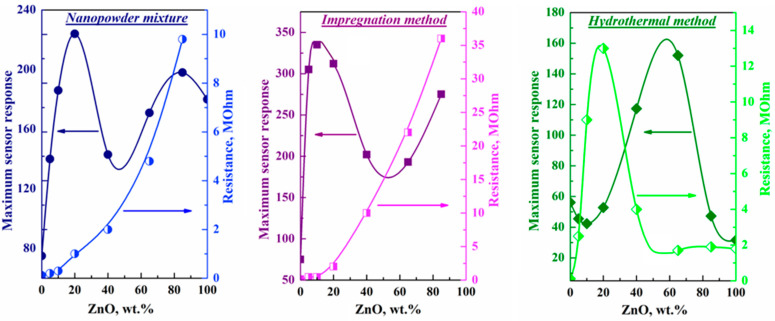
Effect of ZnO concentration on the maximum sensor response to 1100 ppm H_2_ and resistance of composites prepared by mixing powders, impregnation methods, and hydrothermal methods.

At the same time, as can be seen from the experimental data shown in Figure 4, the character of the dependence of the ZnO-In_2_O_3_ composite conductivity on the concentration of zinc oxide is largely determined by the synthesis method.

The nature of the change in the resistance of the impregnated composite, depending on the composition, differs little from the situation observed for the mixed composite. Specifically, the resistance in these systems increases sharply with an increase in the ZnO concentration. An increase in resistance is also observed in ZnO-In_2_O_3_ systems when a heterojunction occurs [31,34]. Impregnated and mixed composites contain phases of indium oxide and zinc oxide, i.e., such nanocomposite films, as well as films of individual oxides, have an electronic type of conductivity.

Due to the large difference in the values of the electron work function between In_2_O_3_ and ZnO (4.3 eV and 5.2 eV, respectively), electrons are transferred from In_2_O_3_ to ZnO nanocrystals at contacts between nanocrystals of these oxides. This circumstance should be taken into account when considering the conductivity process in this system using the percolation model [35].

As a result of the percolation transition, the path of the current flow changes. The conducting particles become nanoparticles of zinc oxide rather than indium oxide nanoparticles. Such a transition causes a sharp decrease in conductivity. It is important to note that the percolation transition in the systems investigated depends on the method used for sample preparation and occurs at different concentrations of zinc oxide. While in order to change the current path, the impregnated composite must contain 40 wt.% ZnO, the percolation transition in a composite formed by mixing nanopowders is observed at a concentration of at least 65 wt.% zinc oxide. This difference is mainly because the size of ZnO nanoclusters formed on the surface of indium oxide does not exceed 10–30 nm. This size is several times smaller than that of ZnO nanoparticles in a mixed composite, the average size of which is approximately 70 nm. The specific surface area of ZnO and, consequently, the contact area between ZnO and In_2_O_3_ particles in the composites investigated differ even more.

All this implies that the transition of electrons from indium oxide to zinc oxide in contact with it occurs more efficiently in an impregnated composite than in a mixed composite. As a result of such a transition of conduction electrons, the current in the system will flow through aggregates of zinc oxide molecules with much lower conductivity. This trend also explains the observed decrease in the conductivity of the impregnated composite at a lower concentration of ZnO than in the mixed composite. With ZnO concentrations above 40%, the conductivity of the impregnated composite becomes significantly less than that of a mixed composite of the same composition in which current flows through In_2_O_3_ nanocrystals.

The curve of change in the resistance of a hydrothermal composite is fundamentally different from the curves observed for ZnO-In_2_O_3_ composites formed by mixing nanopowders and impregnation (Figure 4). When discussing the data on the conductivity of hydrothermal composites, it is necessary to consider the fact that in such composites containing up to 20% zinc oxide, individual particles of this oxide are not formed. Indeed, only zinc ions embedded in the crystal structure of indium oxide are present in the system.

The electronic structure of indium oxide changes as a result of the introduction of zinc into the In_2_O_3_ structure, and an increase in In_2_O_3_ resistance is observed due to a decrease in the concentration of conduction electrons in this oxide (Figure 4). The formation of “holes” in the system during the substitution of indium ions in the In_2_O_3_ lattice for zinc ions, which are acceptors, affects the growth of resistance in composites containing up to 20% zinc oxide. In this case, the electrons are compensated by holes generated by the acceptors, and the resistance consequently increases. Thus, the introduction of zinc into the In_2_O_3_ structure leads to the appearance of *p*-type defects in the system, which results in an increase in the resistance of hydrothermal composites prepared from mixtures of oxides containing up to 20% ZnO. A similar result was obtained for Zn-doped In_2_O_3_ systems synthesized by the sol-gel method, for which enhancement of resistance was observed with an increase in Zn to 3 mol.% [33].

In the structure of hydrothermal composites containing more than 20% ZnO, zinc oxide crystals appear, which, on interaction with modified In_2_O_3_ crystals, leads to a sharp decrease in the resistance of the composite. The substitution of In^3+^ ions for Zn^2+^ ions in the crystal structure of indium oxide is accompanied by the appearance of new oxygen vacancies in the system. A further increase in the ZnO concentration makes the structure even more unstable due to the large number of oxygen vacancies formed. This can lead to structural defects, such as the formation of a solid solution in the embedding. The change in the type of solid solution from substitution to introduction, which contributes to maintaining the stability of the cubic structure of In_2_O_3_, is accompanied by a decrease in the resistance of the composite.

It is important to note that the interaction between ZnO-In_2_O_3_ components plays a significant role in changing the structure and conductivity of the composites. This interaction is manifested to the greatest extent in impregnated and hydrothermal composites. In impregnated samples, specific contacts between indium and zinc oxides lead to a redistribution of electrons in the oxides and, as a result, a change in the conductivity of the system. On the other hand, the interaction of components in composites formed by the hydrothermal method is accompanied by the incorporation of zinc ions into the indium oxide lattice and also leads to a change in the phase composition of the system.

### 3.3. Sensor Properties of ZnO-In_2_O_3_ Composites

The sensor properties of ZnO-In_2_O_3_ composites also depend on the synthesis method and the observed differences in the interaction between the components. The character of the dependence of the maximum sensor response to hydrogen on the ZnO concentration in composites obtained by impregnation and mixing commercial oxide powders using screen-printing technology [14] is nearly similar (for example, Figure 4 shows the maximum response of composites to 1100 ppm H_2_).

According to the current generally accepted model of sensor processes in the detection of reducing compounds, the sensor response is determined by two processes: the adsorption of oxygen and the analyzed gas on the surface of the sensor layer and the reaction of adsorbed compounds with oxygen centers O^–^ on this surface. The formation of O^–^ oxygen centers occurs during the dissociation of chemisorbed oxygen on the surface oxygen vacancies of a metal oxide nanoparticle. In the ZnO–In_2_O_3_ composites investigated, oxygen dissociation with the formation of chemisorbed oxygen atoms occurs mainly on the surface of ZnO nanoparticles. This is because ZnO has a higher catalytic activity than In_2_O_3_. The electrical current in both mixed and impregnated composites containing up to 80% ZnO flows through electron-rich In_2_O_3_ nanoparticles. The current flow path is determined not only by the ratio of component concentrations in the composite but, in accordance with the percolation theory, also depends on the size of oxide particles [18,35].

Upon contact between In_2_O_3_ and ZnO nanoparticles, oxygen atoms formed on the surface of ZnO nanoparticles pass to the surface of In_2_O_3_ nanoparticles, capturing conduction electrons and transforming into anion radicals, O-. These anion radicals participate in the detection reaction of reducing compounds, in particular H_2_. The ZnO also catalyzes the dissociation of adsorbed H_2_. Consequently, these nanocrystals are chemical sensitizers of the sensor reaction in the ZnO-In_2_O_3_ composite. The effect of ZnO on the sensor response in the ZnO-In_2_O_3_ nanocomposite can be attributed to the flow of H atoms from ZnO into In_2_O_3_ nanocrystals, where they react with O^−^ and return electrons to the In_2_O_3_ conduction band. The chemical sensitization of the process is most effective in the composite when conducting aggregates of In_2_O_3_ are in contact with ZnO nanocrystals along the entire current path.

On the curve of the dependence of the maximum response of mixed and impregnated composites to 1100 ppm of hydrogen on composition, there are two maxima at zinc oxide concentrations of 15–20 and 85 wt.%. Note that the maximal response to ethanol in the ZnO-In_2_O_3_ system is also observed at 20% ZnO [26]. The maximum sensor response of impregnated composites for all compositions is 30–50% higher than the response of composites obtained by mixing commercial nanopowders.

The main reason for the different sensor activity of the composites, as well as their structural characteristics discussed above, is that the size of zinc oxide nanoparticles in the mixed composite is several times larger than the size of ZnO nanoclusters deposited on the surface of In_2_O_3_ nanoparticles during impregnation. The low bond strength in the lattice of such clusters and their developed surfaces contribute to a decrease in the energy required for the formation of active oxygen vacancies. As a result, the concentration of active centers in the cluster significantly increases, which causes a higher sensor response when detecting hydrogen with an impregnated composite. The role of contacts between zinc and indium oxide nanoparticles having different electronic structures and morphologies was also observed in the detection of ethanol [21,25,28].

In contrast to mixed and impregnated systems, several characteristic areas can be distinguished on the curve of the dependence of the maximum sensor response on the composition of hydrothermal composites ZnO-In_2_O_3_: 0–20 wt.%, 20–85 wt.%, and above 85 wt.% ZnO. These sites differ in the effect of zinc oxide on the sensor response of the composite. Note that a similar pattern was previously observed in SnO_2_-In_2_O_3_ composites [10]. The sensor response to hydrogen is reduced in composites containing up to 20 wt.% ZnO compared with pure indium oxide. According to X-ray data, no crystalline zinc oxide is formed in such composites, and zinc ions are embedded in the structure of indium oxide.

The magnitude of the sensor response of hydrothermal ZnO-In_2_O_3_ composites containing up to 20% zinc oxide is significantly influenced by the fact that during the synthesis of such composites, a change in the phase composition of In_2_O_3_, namely the transition of the cubic to the rhombohedral phase, occurs. Earlier, we found that the introduction of cerium into indium oxide, leading to its phase changes, causes a sharp drop in the sensor response to hydrogen compared with pure indium oxide [36]. The introduction of zinc into cubic indium oxide also leads to an increase in the response to NO_2_ compared with the undoped sample [33].

An increase in zinc oxide concentration from 20 to 85 wt.% leads to a rise in the sensor response to hydrogen by almost two times. In this case, the formation of crystalline zinc oxide particles and the return of a stable cubic phase of indium oxide are already taking place. It should be noted that in mixed and impregnated composites, indium oxide has an exclusively cubic structure. That is, an increase in the sensor response to hydrogen in hydrothermal composites containing from 20 to 85% zinc oxide is due, on the one hand, to the formation of a heterojunction between ZnO and In_2_O_3_ and, on the other, to a decrease in the proportion of the rhombohedral phase of indium oxide.

Note that the composite containing 65% ZnO has the maximum sensor activity. According to the data obtained, only two phases (cubic indium oxide and hexagonal zinc oxide) are formed in such a system, and an increase in the sensory response is associated with chemical sensitization. A decrease in the sensor response of composites containing more than 85% zinc oxide is associated with the interruption of the flow of current between indium and zinc oxides. This is because nanoparticles of crystalline ZnO are formed on In_2_O_3_ during hydrothermal synthesis and, at such a concentration, completely cover the surface of indium oxide. Accordingly, the sensitivity of the nanocomposite decreases, approaching the sensitivity of nanocrystalline ZnO.

**Figure 5:**
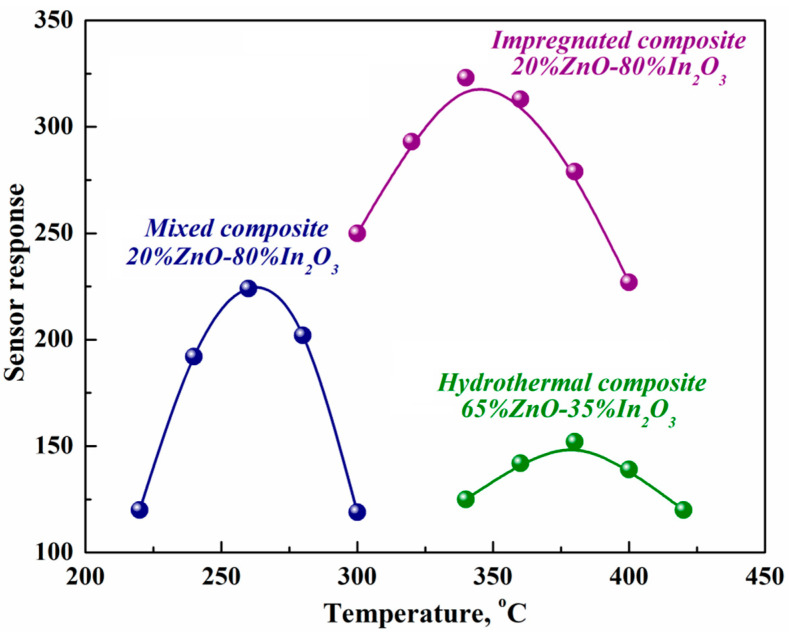
Temperature dependence of the sensor response to 1100 ppm H_2_ of composites obtained by three methods.

As for most metal oxide sensors, including mixed and impregnated ZnO-In_2_O_3_ composites, the temperature dependence of the sensor response of hydrothermal composites to hydrogen passes through the maximum. The maximum temperatures (T_max_) in the detection of 1100 ppm of hydrogen for composites obtained by the hydrothermal method and the impregnation method are 100 and 125 °C, respectively, which are higher than for the mixed composite (Figure 5).

The observed difference in T_max_ is because small ZnO nanoparticles in impregnated and hydrothermal composites contribute to an increase in sensor response, unlike large nanocrystals (larger than 50 nm) contained in mixed composites. This happens because of oxygen dissociation on the surface of nanoparticles, resulting in a large concentration of defects with catalytic activity. Such defects contribute to the formation of oxygen adatoms. The bond of the resulting atoms with defects leads to an increase in the energy required for oxygen desorption and its transition from ZnO particles to In_2_O_3_ particles, which determines the conductivity of the composite. Therefore, the sensitization of the sensor response by ZnO nanoparticles is manifested in such composites at higher temperatures than in mixtures of commercial nanopowders and is more effective due to the high concentration of defects [11].

The main disadvantage of metal oxide sensors is their insufficient selectivity with respect to cross-sensitive gases. In this regard, we studied the influence of the method of obtaining ZnO-In_2_O_3_ composites on their selectivity in the detection of H_2_ with respect to CO. Table 1 shows the values of the sensor response of the composites, with the maximum response to hydrogen upon detection of 1100 ppm H_2_ and CO.

The data presented show that although the composite synthesized by the hydrothermal method has the smallest value of the maximum response to both gases, its selectivity, i.e., the S_H2_/S_CO_ ratio, significantly exceeds the selectivity of composites obtained by mixing oxide nanopowders or by the impregnation method. Such a difference in the selectivity of the samples obtained by different methods is probably due to the change in the structures of the resulting composites and, as a consequence, the sorption activity of the metal oxides included in their composition. The high selectivity of the hydrothermal composite may also be associated with the formation of *p*-type defects [37].

## 4. Conclusions

The analysis of the properties of nanostructured ZnO-In_2_O_3_ composites indicates a significant influence of the method of their formation on the phase composition, structural characteristics, conductivity, and sensor properties in the detection of hydrogen with such materials. The methods of mixing commercial nanopowders and impregnation lead to the formation of two-phase crystalline composites, regardless of the concentration of the components.

In the impregnated composites, the particle size of zinc oxide was smaller than in mixed systems. In turn, in hydrothermal composites, the phase composition is significantly influenced by the concentration of zinc oxide in the composite. Obviously, with any method of composite preparation, the dissociation of oxygen molecules on catalytically active ZnO nanoparticles coupled with further transfer of oxygen atoms to electron-rich In_2_O_3_ nanoparticles and their capture of electrons from these nanoparticles work in the sensor process.

A very important role is played by the interaction between the components of composites due to specific contacts in the process of their synthesis. This factor concerns hydrothermal synthesis to the greatest extent. Specifically, this factor refers to the doping of nanoparticles—the transition of metal ions of one component into nanoparticles of another component with the embedding of metal ions of one oxide into the crystal lattice of another metal oxide.

In the mixed and impregnated ZnO-In_2_O_3_ composites we investigated, contacts between nanoparticles of the oxides led to electron transfer from In_2_O_3_ to ZnO and a gradual increase in the resistance of the composites with a rise in the ZnO concentration in the system. Along with this, in hydrothermal composites containing up to 20 wt.% ZnO, the interaction of nanoparticles leads to a change in the phase composition and structure of In_2_O_3_ particles as a result of the embedding of zinc ions into In_2_O_3_. The maximum resistance in this case is achieved in the system containing 20 wt.% ZnO.

The change in the structure of composites, as well as the different particle sizes, result in the composites formed by the impregnation method having the maximum sensor response to hydrogen. The reason for the relatively low response of hydrothermal composites may be a change in the phase state of indium oxide during hydrothermal synthesis. Unlike mixed and impregnated composites containing only the cubic form of indium oxide, hydrothermal synthesis results in the transition of the cubic form of In_2_O_3_ to a rhombohedral form. This transition probably has a significant effect on the interaction between metal oxides and, consequently, on the sensor process.

## Figures and Tables

**Table 1:** Selectivity and sensitivity in detection of 1100 ppm of H_2_ and CO composites synthesized by three different methods.

	Mixed Composite 20%ZnO-80%In_2_O_3_	Impregnated Composite 20%ZnO-80%In_2_O_3_	Hydrothermal Composite 65%ZnO-35%In_2_O_3_
S_H2_	224	323	152
S_CO_	113	92	11
S_H2_/S_CO_	1.98	3.5	13.8

## Data Availability

The data presented in this study are available on request from the corresponding author.
